# Lutetium-177 Prostate Specific Membrane Antigen Therapy in a Patient With Double Malignancy and Single Functioning Kidney: A Case Report

**DOI:** 10.7759/cureus.36938

**Published:** 2023-03-30

**Authors:** Zikriya Naeem, Umal Baneen Zahra, Muhammad Numair Younis, Irfan Ullah Khan, Abubaker Shahid

**Affiliations:** 1 Nuclear Medicine and PET Imaging, Institute of Nuclear Medicine & Oncology (INMOL) Cancer Hospital, Lahore, PAK; 2 Allied Health Science, FMH College of Medicine and Dentistry, Lahore, PAK; 3 Radiopharmacy, Institute of Nuclear Medicine & Oncology (INMOL) Cancer Hospital, Lahore, PAK; 4 Medical Oncology and Radiotherapy, Institute of Nuclear Medicine & Oncology (INMOL) Cancer Hospital, Lahore, PAK

**Keywords:** rlt safety, renal cell carcinoma, single functioning kidney, renal toxicity, 177lu psma-617, metastatic castration resistant prostate cancer

## Abstract

Lutetium-177 labeled with 617 types of Prostate Specific Membrane Antigen (^177^Lu PSMA-617) Radio-ligand Therapy (RLT) is an emerging modality of choice for the treatment of metastatic castration-resistant prostate carcinoma (mCRPC). After it is administered intravenously, it is excreted primarily through the kidneys. Physiological excretion and concomitant expression of PSMA receptors on renal tissues are associated with potential renal toxicity, a matter of concern while treating patients with multiple doses of RLT. There are published articles that have demonstrated the safe use of ^177^Lu PSMA-617 in patients with bilateral fair-functioning kidneys; however, only a single study has been published that has evaluated its safety in patients with solitary-functioning kidneys. The uniqueness of this case report lies in the fact that we have documented the renal safety profile of ^177^Lu PSMA-617 therapy after multiple doses in a patient who presented with double malignancy (metastatic castration-resistant prostate carcinoma and left renal cell carcinoma) and had a single-functioning right kidney.

## Introduction

Prostate cancer is amongst the most commonly reported malignancy in men worldwide with an incidence of 5%-6% in Pakistan [1]. The patients are identified with mCRPC when there is disease progression despite multiple treatments that include radical prostatectomy and radiotherapy followed by androgen deprivation therapy, hormonal (Enzalutamide and Abiraterone), and chemotherapy (Docetaxel and Cabazitaxel). In such patients, PSMA-targeted radionuclide therapy such as ^177^Lu-PSMA-617 is considered to be a viable therapeutic option [2].

^177^Lu is a reactor-produced radionuclide, which emits Beta (β) radiations of medium energies (490 keV with a maximum of 0.5 MeV) along with low energy Gamma (γ) radiation of 208 keV. It has a half-life of 6.73 days and a tissue penetration power of <2mm [2].

As PSMA is physiologically and specifically expressed in the apical epithelium of proximal renal tubules, possible nephrotoxicity is always suspected as each kidney is exposed to an average absorbed radiation dose of 0.39-0.99 Gy/GBq individually [3] as reported on post-therapy dosimetry.

In the case of a single kidney, it is theoretically acceptable to assume that a higher amount of radiation burden will be disposed to the solitary kidney thereby questioning the tolerability and nephrotoxicity of ^177^Lu PSMA-617 therapy in such patients. Considering the mentioned facts and concerns, our case report is written with the objective to document the practical evidence of the renal safety profile of radionuclide therapy in a patient with dual malignancy and single functioning right kidney.

## Case presentation

A 69-year-old gentleman, diagnosed case of metastatic castration-resistant prostate carcinoma (mCRPC), was referred to the Nuclear Medicine Department at the Institute of Nuclear Medicine and Oncology (INMOL) in August 2021 for ^177^Lu PSMA-617 RLT. The patient presented with widespread skeletal metastasis documented on the bone scan and rising PSA levels despite receiving multiple lines of treatment in the past.

His medical record showed that he was diagnosed with prostate adenocarcinoma of acinar type and a Gleason Score of 7 on a trans-perineal biopsy in March 2017. The patient underwent a radical prostatectomy in April 2017 after the imaging workup including an MRI scan of the pelvis, whole body bone scan and CT scan of the thorax showed that the disease was limited to the prostate gland. Other co-morbidities identified in the clinical history were osteoarthritis, diabetes mellitus, hypertension, and ischemic heart disease Later, the patient received hormonal and androgen deprivation therapy (ADT) over a period of 30 months.

**Figure 1:**
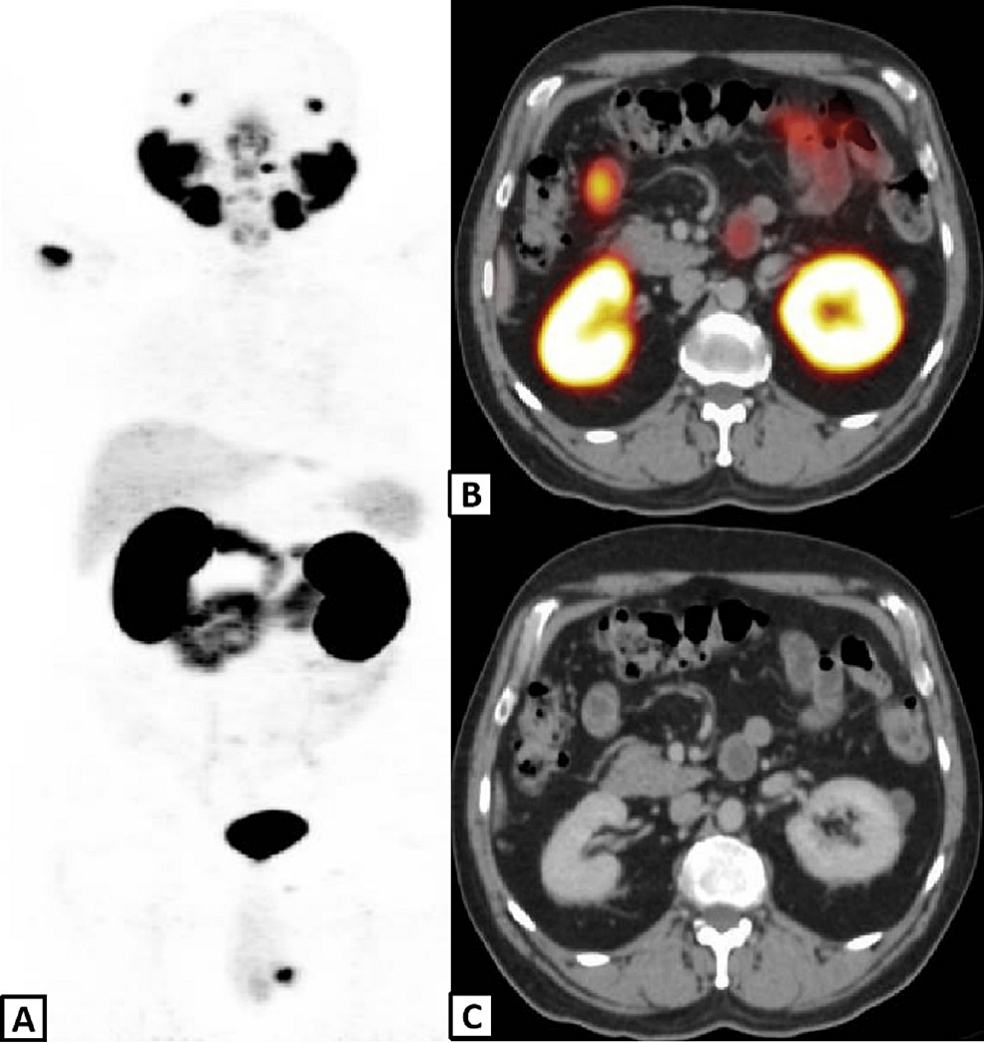
PSMA PET-CT scan Maximum intensity projection (A), fused axial PET-CT (B) and axial CT (C) slices show focal osseous metastasis in right humerus and exophytic mass in left kidney.

Galium-68 (^68^Ga) PSMA PET-CT was performed in 2020 that showed metastatic skeletal deposits and focus of mild PSMA avidity in the left kidney corresponding to mixed density exophytic mass measuring 8mm (Figures 1A-1C). The degree of uptake in the renal mass was much lower as compared to reference organs including the parotid gland and liver and thus was not considered significant as far as the PET scan portion of the PET-CT study.

The region of mild focal uptake corresponding to exophytic mass in the left kidney was subjected to CT guided biopsy. The histopathological diagnosis was consistent with renal cell carcinoma (RCC), a second primary malignancy. Consequently, the patient underwent left radical nephrectomy in March 2020.

In January 2021, the PSA level started rising while the patient was receiving oral therapy with Enzalutamide and Corticosteroids. This relapse of the disease was treated with eight cycles of chemotherapy with the last session in August 2021. Further treatment with the chemotherapy was stopped as serial PSA levels showed a fresh rise during the last two cycles of the chemotherapy.

**Figure 2:**
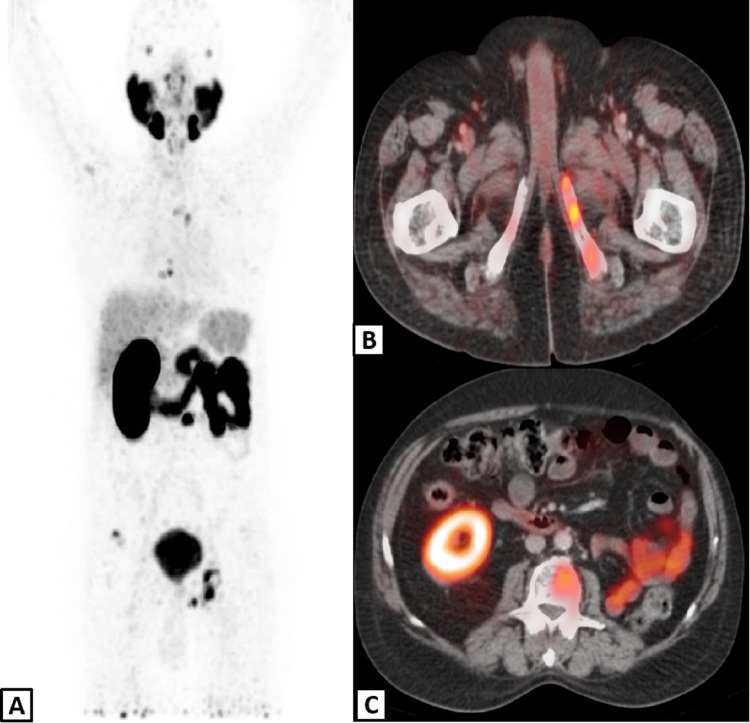
PSMA PET-CT scan Maximum intensity projection (A) and fused axial PET-CT slice (B) demonstrate multiple osseous metastases. The left kidney is not visualized in fused axial PET-CT slice at kidney level (C) due to nephrectomy.

As per pre-requisites of Radioligand Therapy (RLT), the current status of disease and functional status of the single right kidney were evaluated by ^68^Ga-PSMA PET-CT and ^99m^Tc-DTPA renal scans respectively. The PSMA PET-CT performed in August 2021 showed widespread PSMA avid osseous metastasis with highest PSMA avidity reported in sclerotic focus of L4 vertebrae with Standard Uptake Value Max (SUVmax) of 16.33 however no visceral or nodal metastasis were appreciated (Figures 2A-2C).

**Figure 3:**
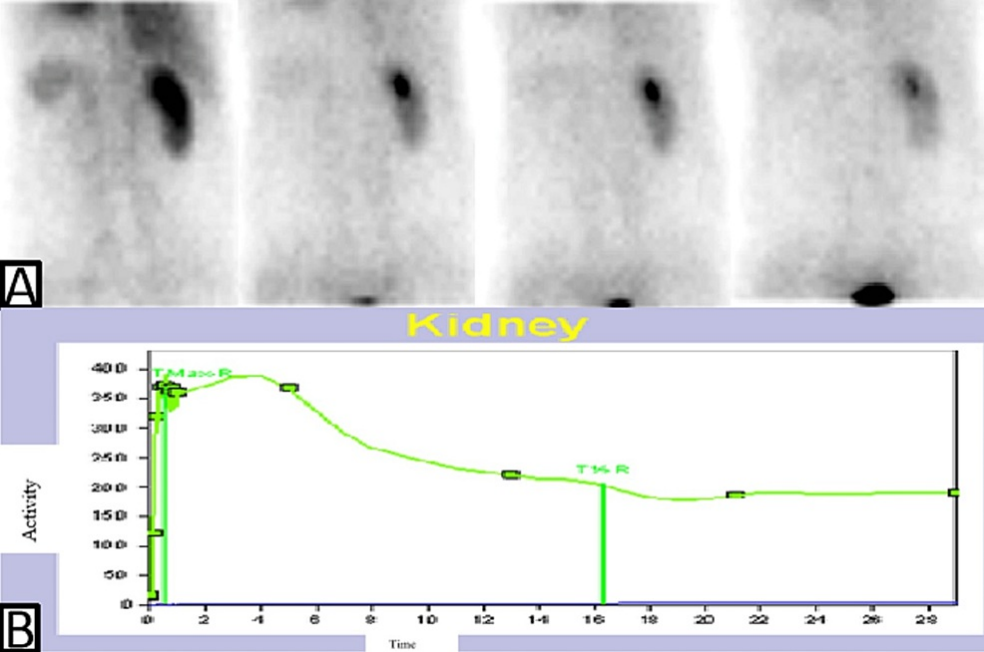
Dynamic renal scan The selected frames from dynamic renal study (A) demonstrate well preserved right kidney function and the left kidney is not visualized due to nephrectomy. The right kidney renogram (B) is showing normal clearance pattern without any evidence of obstruction.

The ^99m^Tc-DTPA renal scan done on 1st September 2021 that showed a fair functional status of right kidney with a GFR of >50mL/min/1.72m^2^ (Figures 3A, 3B).

The case was presented in our Institutional Multidisciplinary Board where the patient’s history, disease extent, performance status and co-morbidities were discussed according to the inclusion criteria as set by the established procedural guidelines of European Association of Nuclear Medicine (EANM) [4] and benefit to risk ratio of administering the radionuclide therapy and its dose was discussed.

Given the patient’s disease progression, adequate GFR of right kidney, optimal baseline creatinine (1.1 mg/dl) and BUN levels (24.39), prior treatment and ECOG score of 1, he was approved for RLT ^177^Lu-PSMA-617 with standard dosage of 6-7 GBq/cycle as per institutional protocol.

The treatment consisted of three cycles of ^177^Lu-PSMA-617 with a mean cumulative dose of 19.5 GBq at an interval of 8 weeks. For renal protection, the blood renal parameters consisting of serial creatinine, BUN and eGFR levels were closely monitored prospectively after each dose at a regular fixed interval of 2, 4 and 8 weeks post therapy, Furthermore, the patient was also properly sensitized to ensure optimum oral hydration (2-3 liters of water per day) and as an additive cover, amino acids analogue regime (Tab. Ketosteril twice a day) was also given for 15 days after each cycle of therapy.

**Figure 4:**
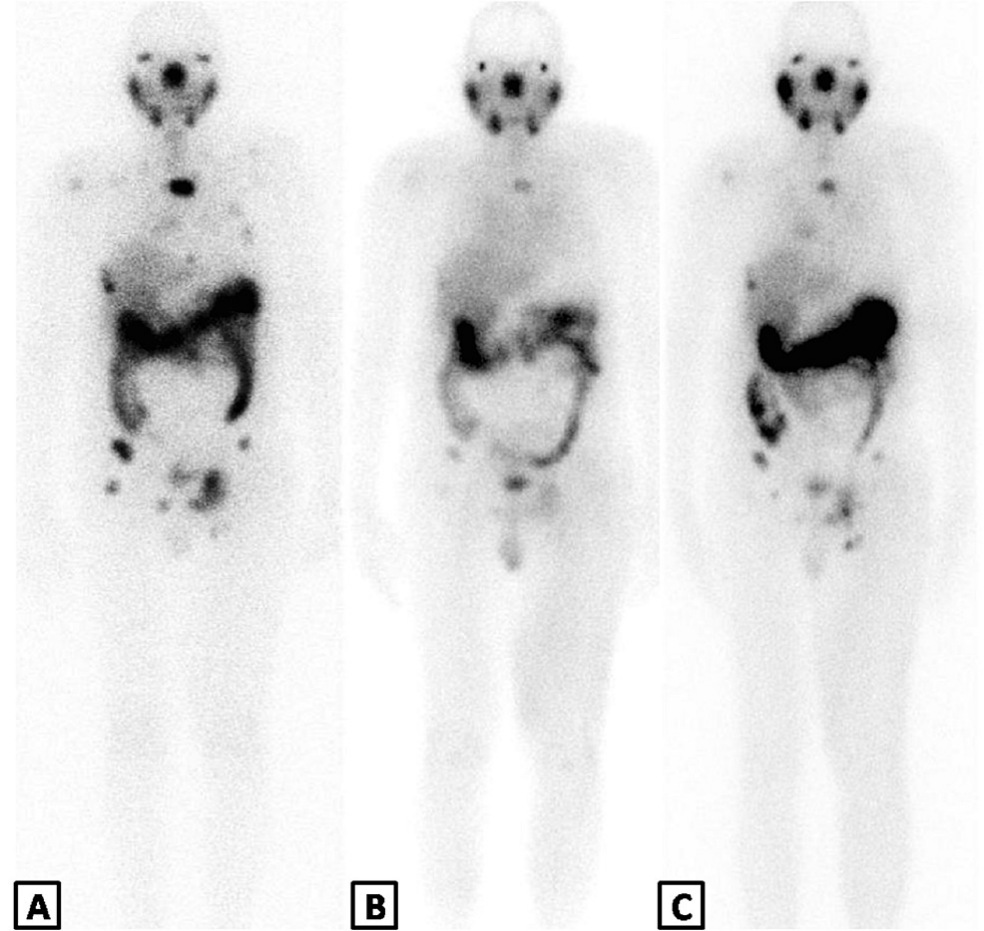
PSMA post therapy whole body scans The post therapy scans were performed at 72 hours after first (A), second (B) and third (C) doses of RLT between September 2021 and January 2022. An increase in number of abnormal sites and degree of uptake can be identified on image C as compared to B, indicating disease progression.

The course of three doses of ^177^Lu- PSMA -617 was completed on January 25, 2022 and follow up RFTs of patient, dated February 2, 2022, showed normal values of creatinine and eGFR levels (0.9mg/dL and >60 mL/min, respectively) and no acute nephro-urological complains were reported. The post therapy ^177^Lu-PSMA scans are exhibited in Figures 4A-4C.

**Figure 5:**
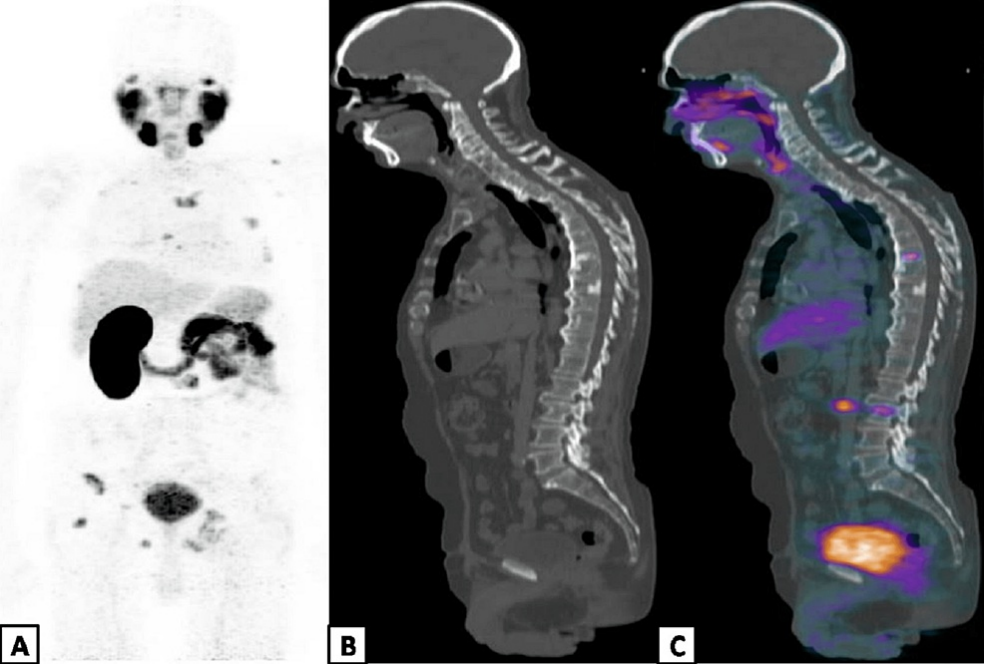
PSMA PET-CT scan Maximum intensity projection (A), sagittal CT (B) and fused sagittal PET-CT (C) demonstrated multiple osseous metastases.

However, the PSA levels demonstrated a sharp rise from 9.4 to 30.1 ng/mL between January 2022 and March 2022 with new sites of bony pains. ^68^Ga-PSMA PET-CT was performed in March 2022 to evaluate the status of the disease before administering another dose of ^177^ Lu- PSMA -617 that shows multiple avid osseous metastases (Figures 5A-5C). 

**Figure 6:**
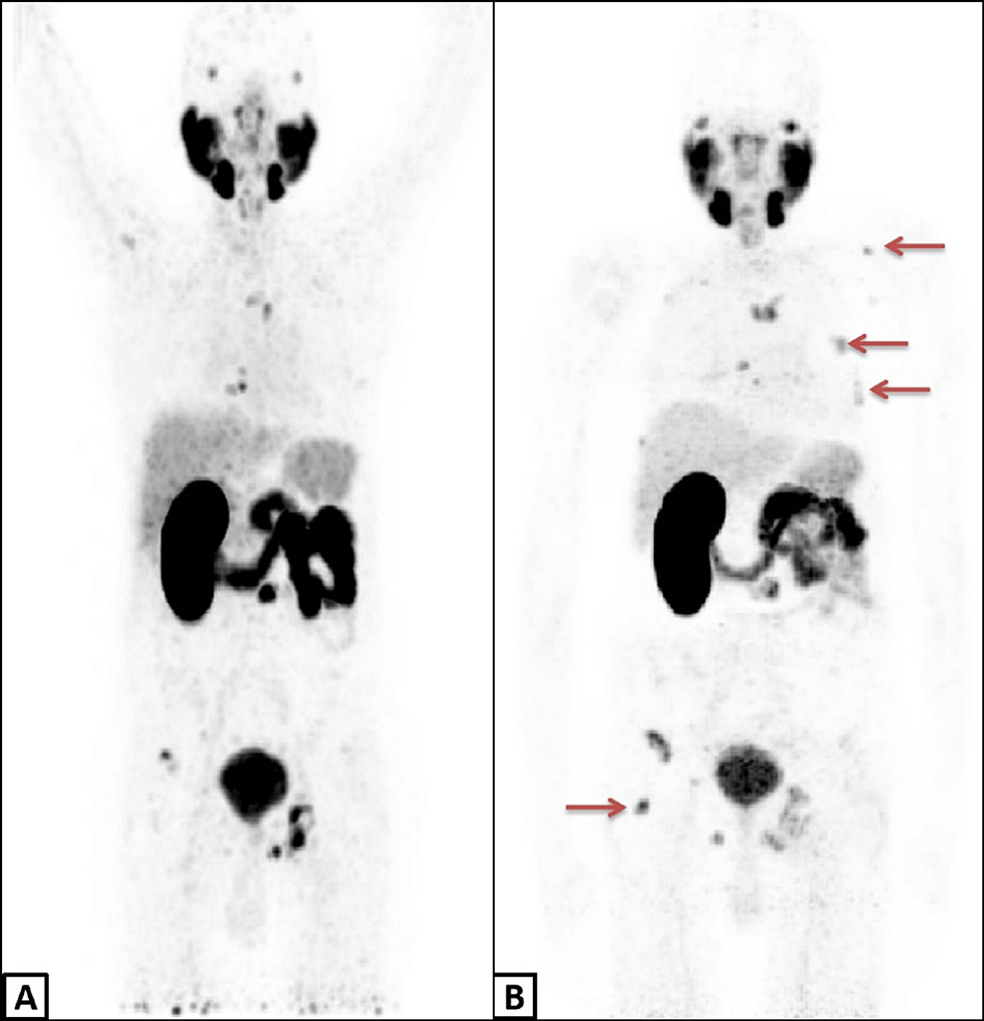
Pre and Post therapy images of PSMA PET-CT scans Maximum intensity projection (MIP) images of PSMA PET-CT scans before (A) and after (B) Lu-177 PSMA therapy demonstrating new metastatic sites marked by red pointer.

As compared to the previous PET-CT scan, the current study demonstrated new osseous deposits showing mild to moderate avidity of tracer and recurrence at some of the previous sites of disease resolution thus indicating disease progression (Figures 6A, 6B).

The findings of PET-CT scan were in agreement with last whole body scan that was performed in January 2022 after administration of third dose of ^177^Lu-PSMA-617. The patient was counselled about lack of therapeutic benefit of further treatment with ^177^Lu-PSMA-617 and was referred to the medical oncology department for consideration of treatment with immunotherapy.

## Discussion

To our knowledge, this is the first case that has highlighted the expressivity of PSMA in two different malignancies in a single patient while documenting the renal tolerability and safety profile of ^177^Lu PSMA-617 therapy despite having a single functioning kidney.

PSMA is a 750 amino acid type II transmembrane glycoprotein with folate hydrolase activity which plays a role in cell migration, cell survival, and proliferation. It is overexpressed (approximately 1,000 times higher) during prostate carcinoma [5]. However, it is not entirely prostate-specific and is expressed physiologically in other non-prostatic cells/tissues too such as the small intestine, proximal renal tubules, and salivary and lacrimal glands [6,7].

Such presence of PSMA receptors in non-target organs has always highlighted and questioned the potential radiation dose-associated side effects and safety profile of ^177^Lu-PSMA-617 radionuclide therapy. Owing to renal excretion of the pharmaceutical and the presence of PSMA receptors in renal tubular structures, potential specific binding and internalization of radioactive ^177^Lu-PSMA molecules in kidney tissues impose a significant risk of nephrotoxicity.

With a reported radiation exposure dose of 0.39-0.99 Gy/GBq for each kidney [3] and theoretically 0.78-1.87 Gy/MBq in the case where a single kidney is present, optimal functioning of the kidneys is a major factor to be ruled out prior to RLT. ^99m^Tc-Diethylene triamine penta-acetic acid (DTPA) renal scan, as performed in our patient, has an established high sensitivity and effectiveness in the evaluation of the dynamic functional status of the kidney(s) and GFR estimation [8].

In a published study, Zhag et al. [9] documented the renal safety of ^177^Lu-RLT in 16 patients carrying single-functioning kidneys. The patients included in the study of Zhag had metastasis in lymph nodes and viscera and demonstrated renal abnormalities such as hydronephrosis and renal atrophy for which ^99m^Tc-MAG-3 renal scintigraphy was used. The authors have documented fair renal tolerability of ^177^Lu-RLT in their patients. However, the current report presented a different scenario. The patient under discussion had left total nephrectomy due to a second malignancy and the ^99m^Tc-DTPA renal scan was used for GFR estimation, contrary to the earlier-mentioned study. Lack of nodal metastasis and visceral deposits signify a low burden of metastatic disease in this patient to accumulate administered PSMA, thereby potentially exposing the solitary kidney to a higher quantity of ^177^Lu-PSMA-617 due to physiological causes.

The ability of ^68^Ga-PSMA PET-CT has also been documented in detecting a second malignancy at unsuspected sites. The evidence of pathological expressivity of PSMA for diagnosis of other primary carcinomas such as RCC has been highlighted in our case. Muselaers et al. [10] in their review of published studies of Spatz et al., Demirci et al., Rhee et al., and Sawicki et al. have reported the sensitivity of 92.11% and a positive predictive value (PPV) of 97.22% of ^68^Ga PET-CT in the diagnosis and staging of RCCs. Furthermore, in the most recent report to date, Tariq et al. [11] have described an intra-patient comparison of PSMA and FDG PET/CT in 11 patients and have documented the superior accuracy of PSMA PET imaging when compared to conventional imaging in RCC.

In concordance with the published data, the reported mass with SUVmax 4.2 of the left kidney on the PET scan of 2020 raised suspicion of the possibility of a second malignancy, which turned out to be a true positive indicating the high accuracy of ^68^Ga-PSMA PET CT in non-prostate neoplasms.

## Conclusions

In the conclusion, the current study has highlighted the safety of ^177^Lu PSMA-617 in patients bearing a single-functioning kidney despite multiple doses of RLT. It appears that the nephrotoxic potential of RLT may be over-estimated particularly in the patients of advanced prostate carcinoma receiving palliative treatment. Dynamic renal functions and renal serum profile however require to be evaluated prior to therapy. This study also emphasizes the importance of unsuspected and incidental findings on PSMA PET-CT scans. The incidental finding of abnormal uptake of PSMA in an unusual site or at a location seemingly unrelated to prostate carcinoma requires due consideration for a second malignancy or sinister disease process. All such findings require further evaluation through histological evaluation or follow-up imaging.
